# GC–MS Profiling and In Vitro and Silico Evaluation of Biological Propensities of Saudi Cultivar of Sugar Apple (*Annona squamosa* L.): A Preliminary Multidimensional Approach for the Development of Nutraceuticals

**DOI:** 10.1002/fsn3.70723

**Published:** 2025-09-24

**Authors:** Hanan Y. Aati, Renad Al‐Arifi, Chitchamai Ovatlarnporn, Khloud AlYami, Abdul Rauf, Abdul Basit, Huma Rao, Maria Batool, Kashif ur Rehman Khan

**Affiliations:** ^1^ Department of Pharmacognosy College of Pharmacy, King Saud University Riyadh Saudi Arabia; ^2^ College of Pharmacy King Saud University Riyadh Saudi Arabia; ^3^ Department of Pharmaceutical Chemistry, Faculty of Pharmaceutical Sciences Prince of Songkla University Hat Yai, Songkhla Thailand; ^4^ Drug Delivery System Excellence Center, Faculty of Pharmaceutical Sciences Prince of Songkla University Hat Yai, Songkhla Thailand; ^5^ College of Pharmacy The University of Health Sciences Lahore Punjab Pakistan; ^6^ Department of Pharmaceutical Chemistry, Faculty of Pharmacy The Islamia University of Bahawalpur Punjab Pakistan

**Keywords:** ADME, *Annona squamosa*, antibacterial, anti‐oxidant, GC–MS, molecular docking, thrombolytic

## Abstract

*Annona squamosa L.*, commonly known as sugar apple or custard apple, is a well‐known medicinal plant recently introduced for cultivation in Saudi Arabia. However, the pharmaceutical potential of the Saudi cultivar remains unexplored. This study aimed to evaluate the phytochemical composition and biological activities of the ethanolic extract of Saudi *A. squamosa* fruit (EASF) through comprehensive in vitro and in silico analyses. Phytochemical screening revealed a high content of phenolics, flavonoids, and tannins.GC‐MS analysis identified 34 bioactive compounds, including fatty acid esters and terpenoids. EASF exhibited strong anti‐oxidant activity, particularly in the ABTS assay, and demonstrated significant thrombolytic potential (90.21% ± 0.90%) along with moderate hemolytic activity (14.1% ± 1.01%). Notably, the extract showed effective antibacterial activity against *Staphylococcus epidermidis* with an inhibition zone of 11 mm at 150 mg/mL. Enzyme inhibition assays revealed potent activity against urease (95.65%), tyrosinase (85.50%), and α‐amylase (47.88%). In silico molecular docking supported these findings, with selected phytoconstituents showing strong binding affinities toward the target enzymes. ADMET profiling further confirmed their drug‐like properties. These results suggest that the Saudi cultivar of *A. squamosa* possesses significant therapeutic potential and may serve as a promising candidate for developing nutraceuticals and pharmaceutical agents.

## Graphical abstract

Highly nutritious and medicinally important plant *Annona squamosa* L. (Sugar apple) showed high levels of functionally important polyphenolic contents. The ethanolic extract exhibited strong antioxidant, thrombolytic, antibacterial, and enzyme inhibition activities. GC–MS profiling showed compounds with drug‐like properties and potential theoretical interaction with biologically critical enzymes.

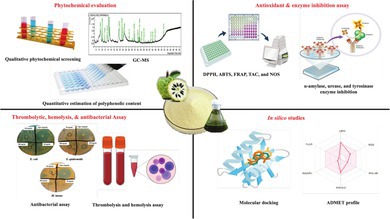

## 1 Introduction

The tropical, endemic species *Annona squamosa L*., belonging to (Annonaceae), commonly known as sugar apple, sweetsop, or custard apple, is widely distributed in America, West Indies, Ecuador, Brazil, Bermuda, Peru, India, the Bahamas, Mexico, and Egypt. It is known for its delectable fruits. The tree starts as a little seedling and develops to a height of 8 m. Its broad, haphazard branches have thin leaves and bark that is either light brown or brown (Kumar et al. 2021). Numerous studies and publications have demonstrated the therapeutic properties of every portion of *A. squamosa* . Spinal disorders and depression are treated internally using roots. Bark has an impressive astringent reputation. Ayurveda regards fruits as an excellent tonic since they improve blood quality, may be an expectorant, strengthen muscles, chill the body, reduce burning sensations and biliousness, calm the heart, and ease vomiting (Varadharajan et al. 2012). This plant's mature fruits are used to accelerate suppuration around malignant tumors. The powdered, dried, unripe fruit is used to kill rodents. The seeds are toxic and bitter. Fish poison and pesticides are made from powdered seeds (Pandey and Barve 2011). The chemical composition of *A. squamosa* has demonstrated its potential as a valuable source of flavoring and nutraceuticals. Previous research has revealed that the fruit is a rich source of bioactive chemicals, which might be utilized to increase the health benefits of value‐added goods and other culinary applications (Bhattacharya and Chakraverty 2016).

Recently, the species has been introduced for cultivation in Saudi Arabia. The geographical differences hugely impact plant species' chemical composition and pharmacological properties. Despite its well‐established traditional uses and reported phytochemical richness, no comprehensive scientific data exist on the chemical composition and pharmacological potential of *A. squamosa* cultivated in Saudi Arabia. The lack of such data limits the ability to evaluate its suitability for medicinal or nutraceutical development in this region, where environmental and geographical differences may significantly influence its bioactive profile. Therefore, it is required to assess every cultivar of a species that has grown in various geographical regions. To the best of our knowledge and literature search, the Saudi cultivar of the species has yet to be explored for its chemical and biological properties. Therefore, the current study was designed to explore the chemical properties through polyphenolic quantification, GC–MS profiling, and pharmacological properties using in vitro anti‐oxidant assays, thrombolytic, hemolytic, antibacterial, and enzyme inhibition assays. At the same time, the compounds tentatively identified in GC–MS analysis were subjected to in silico computational studies. The study design is inspired by sugar apple's versatile properties, including antioxidant, antibacterial, and antidiabetic. Therefore, in this study, we employed four different methods to evaluate the antioxidant potential of the fruit extract. Clinically significant antibacterial strains, namely, *Staphylococcus epidermidis, Micrococcus luteus*, and *Escherichia coli*, were used to assess the antibacterial potential of the fruit extract. At the same time, three different enzymes, α‐amylase, urease, and tyrosinase, were recruited to evaluate enzyme inhibition activity. Furthermore, the toxicity insights were assessed by hemolytic and thrombolytic activity evaluation. This study will provide a way for nutraceutical industries to develop sugar apple‐based products.

## 2 Materials and Methods

### 2.1 Chemicals

Folin ciocalteu reagent, gallic acid, anhydrous sodium carbonate, quercetin, aluminum chloride, sodium nitrite, sodium Hydroxide, tannic acid, ascorbic acid, DPPH sol., ABTS, potassium per sulfate, DMSO, sulfuric acid, sodium phosphate, ammonium molybdate, acetate buffer, ferric chloride, hydrochloric acid, TPTZ, sodium nitroprusside, sulphanilamide, phosphoric acid, naphthyl ethylenediamine dihydrochloride, sodium nitrile, sodium phosphate buffer, sodium chloride, di‐nitro salicylic acid, starch, iodine reagent, acarbose, thiourea, urease, phosphate buffer, aqueous urea, phenol, sodium hypochlorite, streptokinase inj., L‐dopa, tyrosinase enzyme, kojic acid, L‐tyrosine, barium chloride, nutrient broth, agar media, water, methanol, and ethanol were purchased from Sigma Aldrich, Louis, MO 63103, USA.

### 2.2 Preparation of the Plant Extract

Fruits of the *A. squamosa* were bought in June 2022 from a Jazan, Saudi Arabia, farm. At King Saud University's College of Science, Dr. Rajakrishnan Rajagopal recognized these fruits. Every fruit weighing 500 g was gathered, cleaned, and then chopped into little pieces. They were then allowed to dry in the cold air and crushed to create a powder. Then, we immersed each powder in 250 mL of 80% ethanol (×4), stirring often for 3 days and filtering. The soaking procedure was carried out four times to ensure that all active chemicals were removed. Following that, the alcoholic extracts were gathered and dried using a rotary evaporator to produce dark green residues, which were then stored in a dark container in the refrigerator for additional biological and phytochemical examinations.

### 2.3 Phytochemical Analysis

#### 2.3.1 Preliminary Phytochemical Analysis

Using a qualitative phytochemical examination of EASF, the presence of primary and secondary metabolites, that is, amino acids, carbohydrates, lipids, proteins, reducing sugar, flavonoids, cardiac phenols, glycosides, tannins, saponins, steroids, and alkaloids, was investigated using the protocols reported in the literature (Rao et al. 2023).

#### 2.3.2 Total Bioactive Contents

##### 2.3.2.1 Total Phenolic Content (TPC)

The total phenolic content (TPC) was determined using the Folin–Ciocalteu method as previously described (Ahmad et al. 2022). A calibration curve was prepared using gallic acid standards at 5–50 μg/mL concentrations. An aliquot of 125 μL of the sample solution (1 mg/mL) was transferred into an Eppendorf tube, followed by the addition of 500 μL of Folin–Ciocalteu reagent. Subsequently, 375 μL of 7.5% sodium carbonate (Na₂CO₃) solution was added, and the reaction mixture was incubated in the dark for 2 h. The absorbance was measured at 765 nm using a BioTek Synergy HT microplate reader.

##### 2.3.2.2 Total Flavonoid Content (TFC)

The total flavonoid content (TFC) was estimated using the aluminum chloride colorimetric method with minor modifications from previously reported procedures (Ahmad et al. 2022). A standard calibration curve was generated using Quercetin in methanol (5–50 μg/mL). To 0.3 mL of sample solution (1 mg/mL), 1.3 mL of distilled water was added, followed by 0.1 mL of 5% sodium nitrite and 0.1 mL of 10% aluminum chloride. After 5 min, 0.66 mL of sodium hydroxide was added, and after an additional 6 min, 0.8 mL of distilled water was added. Ethanol was used as a blank. Absorbance was measured at 510 nm using a BioTek Synergy HT microplate reader.

##### 2.3.2.3 Total Tannin Content (TTC)

Total tannin content (TTC) was determined using the Folin–Ciocalteu method as previously reported (Tambe and Bhambar 2014). Methanolic quercetin solutions (5–50 μg/mL) were used to construct a standard curve. To 0.3 mL of the sample (1 mg/mL), 1.3 mL of distilled water, 0.1 mL of sodium nitrite, and 0.1 mL of 10% aluminum chloride solution were sequentially added. After 6 min, 0.66 mL of sodium hydroxide was added, followed by 0.8 mL of distilled water. Ethanol was a blank, and absorbance was recorded at 510 nm using a BioTek Synergy HT microplate reader.

#### 2.3.3 Gas Chromatography–Mass Spectrometry Analysis (GC–MS)

GC–MS analysis of the extract was conducted using an Agilent 5977A GC System equipped with an HP‐5MS capillary column (30 m × 250 μm × 0.25 μm; maximum temperature: 350°C) coupled to an Agilent 5977A MSD. High‐purity helium (99.99%) was used as the carrier gas at a constant flow rate of 1.2 mL/min. The injector, transfer line, and ion source temperatures were maintained at 310°C, with an ionization energy of 70 eV. The oven temperature program was as follows: initial temperature 60°C (held for 7 min), increased at 5°C/min to 310°C. The injection volume was 1 μL with a 50:1 split ratio. Full scan mass spectra were recorded from 35 to 650 amu. Compound identification was performed by comparing the retention times and mass fragmentation patterns with those in the NIST‐02 mass spectral library (Aati et al. 2022).

### 2.4 Biological Activities

#### 2.4.1 Anti‐Oxidant Activities

##### 2.4.1.1 Total Antioxidant Capacity (TAC) by the Phosphomolybdenum Method

The phosphomolybdenum method was used to assess TAC as previously described (Untea et al. 2018), with slight modifications. Ascorbic acid (1 mg/mL in 5% DMSO) was used as the standard, and a calibration curve was established with concentrations ranging from 50 to 1000 μg/mL. For each test, 130 μL of the ascorbic acid solution or plant extract (1 mg/mL) was combined with phosphomolybdenum reagent to a final volume of 1 mL. Samples were incubated at 95°C for 90 min, and absorbance was measured at 695 nm (Untea et al. 2018).

##### 2.4.1.2 2,2‐Diphenyl‐1‐Picrylhydrazyl Assay (DPPH)

The DPPH assay followed the established protocols (Aati et al. 2022) with slight modifications. A 0.3 mM DPPH solution was prepared in methanol. To 90 μL of this solution, 90 μL of the test sample was added to each well of a microplate. The mixture was incubated in the dark at room temperature for 30 min, and absorbance was recorded at 517 nm using a BioTek Synergy HT microplate reader.

##### 2.4.1.3 2,2‐ Azino‐Bis(3‐Ethylbenothiazoline) 6‐Sulfonic Acid (ABTS)

ABTS assay was performed according to the literature (Grochowski et al. 2019) with minor modification**s**. Standard calibration curves were established using ascorbic acid concentrations ranging from 5 to 50 μg/mL. After adding 1 mL of ABTS, 1 mL of potassium persulfate, and 1 mL of sample solution, the test tubes were left in the dark for 30 min. A BioTek Synergy HT microplate reader was then used to detect the absorbance at 620 nm.

##### 2.4.1.4 Ferric Reducing Anti‐Oxidant Power (FRAP)

ABTS radical scavenging activity was assessed using a reported method (Grochowski et al. 2019) with minor modifications. Standard curves were generated using ascorbic acid (5–50 μg/mL). Equal volumes (1 mL each) of ABTS solution, potassium persulfate, and the plant extract were mixed and incubated in the dark for 30 min. Absorbance was measured at 620 nm.

##### 2.4.1.5 Nitric Oxide Scavenging (NOS) Assay

NOS activity was determined using a modified protocol (Yen et al. 2001). Sodium nitrite standards (5–50 μg/mL) were used for calibration. To 1 mL of sample solution (1 mg/mL), 0.25 mL of sodium nitroprusside was added and incubated for 2 h at room temperature. Then, 0.5 mL of the incubated solution was mixed with 0.3 mL of Griess reagent. Absorbance was measured at 570 nm using a BioTek Synergy HT microplate reader.

#### 2.4.2 Enzyme Inhibition Activities

##### 2.4.2.1 Tyrosinase Inhibition Assay

The tyrosinase inhibitory activity was assessed using a previously described method (Khan et al. 2024) with slight modifications. In a 96‐well ELISA plate, 20 μL of tyrosinase enzyme, 10 μL of test sample, and 150 μL of phosphate buffer were added and incubated for 10 min. A pre‐read was taken at 480 nm. Then, 20 μL of L‐DOPA substrate was added, and the plate was incubated for 30 min. The final absorbance was measured at 480 nm. Kojic acid and water were used as positive and negative controls, respectively.

$$\begin{array}{c} \text{Inhibition}\%=\left[\left(\text{Absorbance of post read}—\text{Absorbance of}\;\mathrm{pre}-\text{read}\right.\right.\\ \;\left.\left./\text{Absorbance of control}\right)\right]\times 100 \end{array}$$

##### 2.4.2.2 α‐Amylase Inhibition Assay

The inhibitory activity of α‐amylase was assessed using the starch‐iodine method as previously described (Aati et al. 2022), with slight modifications. Briefly, 100 μL of 0.02 M sodium phosphate buffer (pH 6.9), 100 μL of soluble starch solution, 100 μL of plant extract or acarbose (used as a standard inhibitor), and 100 μL of NaCl were mixed and incubated at 37°C for 5 min. After this, 195 μL of α‐amylase solution was added, and the mixture was incubated for 10 min at 37°C. The enzymatic reaction was then terminated by adding 260 μL of 3,5‐dinitrosalicylic acid (DNS) reagent. Subsequently, 100 μL of iodine reagent was added to the wells. Absorbance was measured at 620 nm using a BioTek Synergy HT microplate reader, and the intensity of the resulting color was indicative of the residual starch content in the reaction mixture.

$$\begin{array}{c} \text{Inhibition}\%=\left(\text{absorbance of control}—\text{absorbance of extract}\right.\\ \;\left./\text{absorbance of control}\right)\times 100 \end{array}$$

##### 2.4.2.3 Urease Inhibition Assay

The anti‐urease activity of EASF was evaluated following a previously reported method (Ahmad et al. 2022) with minor modifications. In brief, 10 μL of urease solution, 10 μL of phosphate buffer (pH 7.0), and 10 μL of the test sample were added to each well of a 96‐well microplate and incubated at room temperature for 15 min. Subsequently, 30 μL of aqueous urea was introduced to the reaction mixture, followed by a second incubation for 15 min. After an initial absorbance reading at 630 nm, 50 μL of alkali reagent and 30 μL of phenol reagent were sequentially added. The mixture was then incubated for an additional 90 min at room temperature. Final absorbance was measured at 630 nm using a BioTek Synergy HT microplate reader.

$$\begin{array}{c} \text{Inhibition}\;\left(\%\right)\;\text{of urease enzyme}=100—\left[\left(\text{Absorbance of post read}\right.\right.\\ \;\left.-\text{absorbance of}\;\mathrm{pre}\;\text{read}\right)\\ \;\left./\text{Absorbance of control}\right]\times 100 \end{array}$$

#### 2.4.3 Antibacterial Activity

The antibacterial activity of EASF was estimated using a previously described method in the literature (Aati et al. 2022) with some modifications.

##### 2.4.3.1 Strains of Bacteria

Antibacterial activity was performed against three strains: *Staphylococcus epidermidis, Micrococcus luteus*, and *Escherichia coli* . The Microbiology lab of the Islamia University of Bahawalpur provided these bacterial strains.

##### 2.4.3.2 Agar Well Diffusion

Briefly, bacterial strains were cultured in nutrient broth test tubes and incubated overnight at 37°C. Colonies were collected, vortexed, and suspended in sterile saline solution, adjusting turbidity according to the McFarland standard. After 24 h of incubation, bacterial growth was observed. Mueller Hinton agar plates were inoculated with different bacterial strains by evenly spreading the cultures across the agar surface. A sterile cork borer punched four wells (6 mm in diameter) into each plate. Sterile filter paper discs impregnated with the plant extract (1 mg/mL) or standard antibiotic (ciprofloxacin) were placed into the wells. The plates were incubated at 37°C for 24 h. On the third day, zones of inhibition were measured in millimeters using a digital caliper. Ciprofloxacin served as the positive control.

#### 2.4.4 Thrombolytic Activity

Thrombolytic activity was assessed following a previously reported method (Aati et al. 2022) with minor modifications. Fresh human blood samples were collected and incubated in pre‐weighed Eppendorf tubes at 37°C for 45 min to allow clot formation. After clot formation, serum was carefully removed without disturbing the clots, and the tubes were weighed again to determine clot weight (Wc). 100 μL of plant extract solution (1 mg/mL) was added to each clot‐containing tube. Streptokinase was used as a positive control. The tubes were then incubated at 37°C for 90 min. After incubation, the released fluid was removed, and the tubes were reweighed to determine the weight of the released clot (Wr). The percentage of clot lysis was calculated using the following formula:

$$\text{Percentage of clot lysis}=\left(\mathrm{Wr}/\mathrm{Wc}\right)\times 100$$

where, Wr = released clot weight and Wc = clot weight.

#### 2.4.5 Hemolytic Activity

The hemolytic activity was performed according to the literature (Aati et al. 2022) with some modifications. The initial toxicity of phytochemical substances derived from plant extract was assessed by utilizing a hemolytic activity on human erythrocytes. The erythrocyte suspension was made using human blood that was in good health. Plasma was extracted from the blood after it was centrifuged for 15 min at 4000 rpm. Phosphate buffer (pH 7.4) was used to cleanse the remaining blood. After combining 25 μL of erythrocytes with 975 μL of extract, the mixture was incubated for 90 min at 37°C and centrifuged for 3 min at 2000 rpm. After obtaining the supernatant, the hemolysis's absorbance at 540 nm was calculated using a BioTek Synergy HT microplate reader. Phosphate‐buffered saline was used as the negative control, and 0.1% Triton X‐100 was used as the positive control.

### 2.5 In Silico Activities

#### 2.5.1 Molecular Docking

Molecular docking studies evaluated the binding affinities of selected phytochemicals with key target enzymes. Crystal structures of α‐amylase (PDB ID: 1BLI), tyrosinase (PDB ID: 2Y9X), and urease (PDB ID: 2KAU) were retrieved from the Protein Data Bank. Protein preparation was conducted using Discovery Studio 2021, where water molecules, heteroatoms, and all chains except chain A were removed. Polar hydrogens were added, and the proteins were saved in the PDB format. Bioactive compounds identified from the GC–MS analysis were selected as ligands. Their structures were downloaded in the SDF format from the PubChem database. Using PyRx software, ligands were converted to the ePDBQT format, and energy was minimized using Open Babel. The protein structures were also imported into PyRx, and a grid box was generated to define the docking site. Molecular docking simulations were performed, and binding affinities were calculated. The ligand–protein interactions were visualized using Discovery Studio to analyze hydrogen bonds, hydrophobic contacts, and other key interactions (Tabassum et al. 2022).

#### 2.5.2 ADME Analysis

Using the online SwissADME tool http://www.swissadme.ch/, the ADME properties of the best docked bioactive compounds were evaluated.

#### 2.5.3 Toxicity Evaluation

Using the online program PROTOX II https://tox‐new.charite.de/, the toxicity of the best‐docked compounds was evaluated.

### 2.6 Statistical Analysis

Readings in each experiment were taken in triplicates and expressed as mean ± standard deviation. One‐way ANOVA followed by Tukey's multiple comparison test was applied to determine statistical significance using GraphPad Prism 7.0 software. The *p*‐value < 0.05 was considered statistically significant.

## 3 Results

### 3.1 Phytochemical Analysis

#### 3.1.1 Preliminary Phytochemical Analysis

Phytoconstituents of EASF were assessed using phytochemical profiling tests. A preliminary phytochemical analysis was conducted on EASF. This investigation revealed that proteins and lipids were present in the extracts, including many primary and secondary metabolites. Nonetheless, a small percentage of carbohydrate content was found. The plant was discovered to have specific amounts of amino acids. It was found that extracts containing phenols included alkaloids as secondary metabolites. The plant showed no signs of saponins. The assay reveals the presence of cardiac glycosides while excluding steroids. Table 1 shows that primary and secondary metabolites (i.e., alkaloids, carbohydrates, flavonoids, cardiac glycosides, phenols, proteins, lipids, sugars, and amino acids) are present in the EASF.

**TABLE 1:** Preliminary phytochemical profiling of EASF.

Metabolite	Test	Result
Amino acids	Ninhydrin test	+
Carbohydrates	Molisch's reagent	+
Lipids	Saponification test	+
Protein	Biurette test	+
Reducing sugar	Fehling's test	+
Cardiac glycosides	Keller‐Kiliani test	+
Flavonoids	Ferric chloride test	+
Phenols	Ferric chloride test	+
Saponins	Froth test	−
Steroids	Salkowski's test	−
Tannins	Lead acetate test	−
Alkaloids	Hager's test	+

*Note:* +, Present; −, Absent.

#### 3.1.2 Total Bioactive Content

The results of total bioactive contents are expressed in Table 2. TPC of EASF was calculated as recorded to be 95.5 ± 5 mg of gallic acid equivalent per gram of dry extract. TFC was 126.44 ± 1.66 mg of quercetin equivalent per gram of dry extract, and TTC was 39.33 ± 1.66 mg of tannic acid equivalent per gram of dry extract.

**TABLE 2:** Total bioactive content of EASF.

Plant	TPC (mg GAE/g D.E)	TFC (mg Q.E/g D.E)	TTC mg TAE/g D.E
EASF	95.5 ± 5	126.44 ± 1.66	39.33 ± 1.66

*Note:* All the assays were performed thrice, and results are expressed in mean ± standard deviation.

#### 3.1.3 GC–MS

**FIGURE 1:**
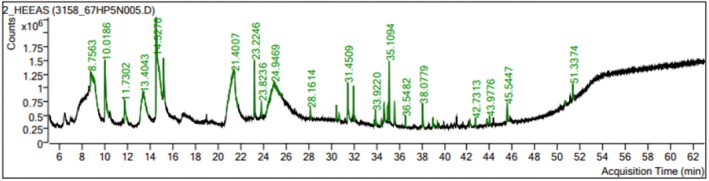
Peaks of identified compounds in EASF by GC–MS.

The GC–MS chromatogram of EASF showed 34 peaks of phytocompounds. Major phytocompounds of GC–MS are kauren‐19‐oic acid, 17 beta‐hydroxy‐5α.‐androstane acetate, isopimaric acid, methyl ester, stigmasterol, and gamma‐sitosterol. The major chemical classes from EASF were fatty acids, sterols, terpenoids, and other bioactive compounds (phenolic compounds, fatty acid esters, and steroids, etc.). Additionally, esters were found in a separate class of phytochemicals with various biological functions. Results are expressed in Table 3, and the peaks are shown in Figure 1.

**TABLE 3:** Phytocompounds identified in the extract of EASF.

Peak no.	Retention time (min)	Area	Compound name	Mol. weight	Mol. formula	Class
1	8.7563	8.129303	3(2H)‐Furanone, 4‐hydroxy‐5‐methyl‐	114.1	C_5_H_6_O_3_	Furans
2	10.0186	4.819964	2,4(1H,3H)‐Pyrimidinedione, 5‐methyl‐	126.11	C_5_H_6_N_2_O_2_	Pyrimidnediones.
3	11.73018	1.979705	oxopyrans	144.12	C_6_H_8_O_4_	Pyranones
4	13.40433	7.407334	L‐Sorbose	180.16	C_6_H_12_O_6_	Ketose sugar
5	14.52757	14.42181	5‐Hydroxymethylfurfural	126.11	C_6_H_6_O_3_	Furans
6	15.18545	1.735572	2‐Decenal, (E)‐	154.25	C_10_H_18_O	Aldehydes
7	21.40065	16.10695	Guanosine	283.24	C_10_H_13_N_5_O_5_	Nucleosides
8	23.22457	1.753408	Beta‐spathulenol	220.35	C_15_H_24_O	Terpenoids
9	23.82362	0.448084	Hexadecane	226.44	C_16_H_34_	Alkanes
10	24.94685	14.32092	1‐Decanamine	157.3	C_10_H_23_N	Amines
11	28.16142	0.349691	Octadecane	254.5	C_18_H_38_	Alkanes
12	30.44532	0.451172	7,9‐Di‐tert‐butyl‐1‐oxaspiro (4,5) deca‐6,9‐diene‐2,8‐dione	276.4	C_17_H_24_O_3_	α, β—unsaturated ketone
13	30.68067	0.329907	Hexadecanoic acid, methyl ester	270.5 g	C_17_H_34_O_2_	Fatty acid methyl esters
14	31.45088	2.228025	Hexadecanoic acid	256.42	C_16_H_32_O_2_	Fatty acid
15	31.98575	0.989568	Hexadecanoic acid, ethyl ester	284.5	C_18_H_36_O_2_	Fatty acid ethyl esters
16	32.09272	0.140083	ICOSANE	282.5	C_20_H_42_	Alkanes
17	33.79897	0.181744	9,12‐Octadecadienoic acid (Z, Z)‐, methyl ester	294.5	C_19_H_34_O_2_	Fatty acid methyl esters
18	33.92198	0.509784	9‐Octadecenoic acid, methyl ester, (E)‐	296.5	C_19_H_36_O_2_	Fatty acid methyl esters
19	34.42477	0.229708	Methyl stearate	298.5	C_19_H_38_O_2_	Fatty Acyls
20	34.6708	0.827736	Oleic acid	282.5	C_18_H_34_O_2_	Fatty acids
21	34.99707	0.424363	Linoleic acid ethyl ester	308.5	C_20_H_36_O_2_	Fatty acid ethyl esters
22	35.1094	1.874069	9‐Octadecenoic acid (Z)‐, ethyl ester	310.5	C_20_H_38_O_2_	Fatty acid ethyl esters
23	35.59613	0.738099	Octadecanoic acid, ethyl ester	312.5	C_20_H_40_O_2_	Fatty acid ethyl esters
24	36.5482	0.367389	Docosane	310.6	C_22_H_46_	Alkanes
25	38.07793	0.954923	9‐Octadecenethioic acid,12‐hydroxy‐, S‐t‐butyl ester	370.6	C_22_H_42_ O_2_S	Thioester
26	38.99255	0.489091	Kauren‐19‐oic acid	302.5	C_20_H_30_O_2_	Diterpinoids
27	39.37232	0.182373	17.beta‐Hydroxy‐5.alpha.‐androstane acetate	318.5	C_21_H_34_O_2_	Steroids
28	42.23922	0.350413	Agathic acid	334.4	C_20_H_30_O_4_	Sesquiterpenoids
29	42.7313	0.324127	Isopimaric acid, methyl ester	316.5	C_21_H_32_O_2_	Sesquiterpenoids
30	43.76895	0.222023	Tetrahydroaraucarolone	338.5	C_20_H_34_O_4_	Diterpinoids
31	43.97755	0.273137	Tetracosanoic acid, methyl ester	382.7	C_25_H_50_O_2_	Fatty acid ester
32	45.54472	0.792998	1‐Undecene‐3,6‐dione, 1‐(2,6,6‐trimethyl1‐cyclohexen‐1‐yl)‐	304.5	C_20_H_32_O_2_	Sesquiterpenoids
33	50.6474	0.177758	Stigmasterol	412.7	C_29_H_48_O	Sterols
34	51.33738	0.669363	Gamma‐sitosterol	414.7	C_29_H_50_O	Sterols

### 3.2 Biological Activities of EASF

#### 3.2.1 Antioxidant Activities

The antioxidant potential was evaluated by FRAP, DPPH, TAC, NOS, and ABTS methods. Table 4 shows the findings of antioxidant assays and their correlation with bioactive contents. The ABTS result suggested that EASF exhibited the highest activity with 319.5 ± 5 mg AAE/g D.E. The results obtained from the EASF were comparable and indicate that this plant possesses good antioxidant potential. Furthermore, a number of compounds were identified from EASF by GC–MS, for their antioxidant activities had previously been characterized. When evaluated for FRAP, the extract's reduction potential showed the maximum reduction potential of EASF to be 186.65 ± 2.76 (mg AAE/g D.E). DPPH results showed activity of 101.95 ± 6.90 (mg AAE/g D.E). Respectively, TAC and NOS showed 217.5 ± 0.41 mg (mg AAE/g D.E) results and 106.40 ± 0.35 (mg AAE/g D.E). Several bioactive phytocompounds with antioxidant activities were also shown by our GC–MS analysis results (s 3), which may have contributed to the extracts' reducing power. Nonetheless, notably elevated levels of TPC and TFC in the EASF could be a factor in the notably elevated anti‐oxidant activities.

**TABLE 4:** Antioxidant potential of EASF.

Plant	ABTS	DPPH	FRAP	TAC	NOS
EASF	319.5 ± 5	101.95 ± 6.90	186.65 ± 2.76	217.5 ± 0.41	106.40 ± 0.35

*Note:* All the assays were performed thrice, and results are calculated as mg AAE/g D.E and expressed in mean ± standard deviation.

#### 3.2.2 Enzyme Inhibition Activities of EASF

EASF's enzyme inhibition activity has also been examined concerning its possible application in cosmeceuticals that could be used for skin‐lightening (with a focus on tyrosinase inhibitory potential), antidiabetic activity (α‐amylase inhibition), as well as anti‐ulcer properties (i.e., urease inhibition). This study used acarbose, kojic acid, and thiourea as a standard to determine the enzyme inhibition activity. The α‐amylase activity result showed that EASF exhibits the α‐amylase inhibitory effect at 47.88% compared to the standard acarbose at 97.16%. The tyrosinase activity result showed that EASF exhibits the tyrosinase inhibitory effect at 85.50% compared to the standard kojic acid at 88.40%. Urease activity results showed that the ethanolic extract exhibits the urease inhibitory effect at 95.65% compared to the standard thiourea at 94.20%. The results of the enzyme inhibition potential of EASF are expressed in Table 5.

**TABLE 5:** Enzyme inhibition % of EASF.

Plant name	Alpha amylase enzyme inhibition	Tyrosinase enzyme inhibition	Urease enzyme inhibition
EASF	47.88	85.50	95.65217391
Standard	97.16^a^	88.40^b^	94.20289855^c^

*Note:* All results are expressed in percentage.

^a^ Acarbose.

^b^ Kojic acid.

^c^ Thiourea.

#### 3.2.3 Antibacterial Activity

**FIGURE 2:**
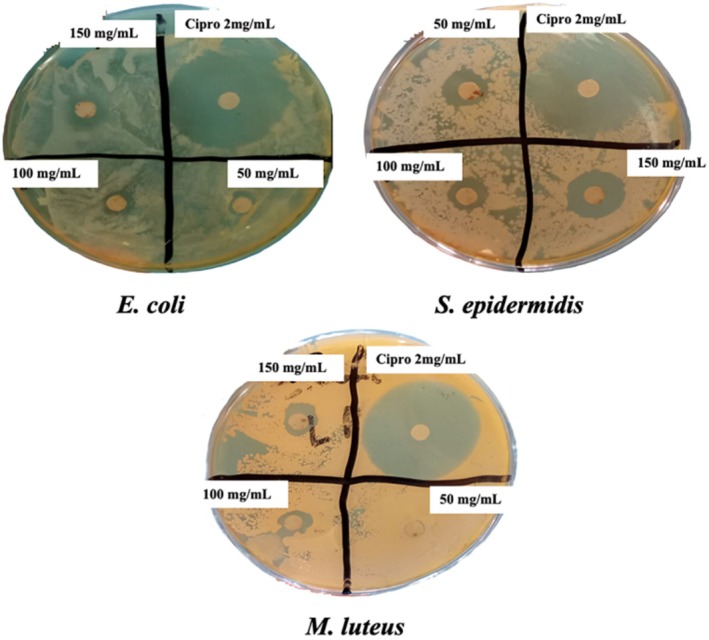
Evaluation of antibacterial activity of EASF in different concentrations (50, 100, and 150 mg/mL). Ciprofloxacin (C) was used at 2 mg/mL. A scale was used to measure the diameter, and the results were expressed regarding the zone of inhibition (mm).

As seen in Table 6 and Figure 2, the extract of EASF was found to have significant activity in evaluating its antibacterial properties. EASF exhibited dose‐dependent antibacterial activity. The maximum activity was seen in the samples with 150 mg/mL, which was not close to the standard antibiotic used in this study (ciprofloxacin). Moreover, *S. epidermidis* was more susceptible than *M. luteus* and *E. coli*. Maximum inhibition zones were seen against *S. epidermidis* (11 mm) compared to ciprofloxacin (22 mm).

**TABLE 6:** Details of the zone of inhibition showed by EASF at various concentrations and ciprofloxacin in antibacterial activity evaluation.

Bacterial strains	50 mg/mL (mm)	100 mg/mL (mm)	150 mg/mL (mm)	Standard ciprofloxacin 2 mg/mL
*S. epidermidis*	8	9	11	22
*M. luteus*	1	5	7	31
*E. coli*	3	4	6	13

*Note:* A scale measured the diameter, and the results were expressed in terms of the zone of inhibition (mm).

#### 3.2.4 Thrombolytic Activity

In evaluating the thrombolytic activity of EASF, the significant activity of the extract was observed, as shown in Table 7. The activity observed in percentage is 90.21% ± 0.90%, which is a significant activity observed compared to the standard of 97.32% ± 1.04%.

**TABLE 7:** Thrombolytic and hemolytic activity of EASF.

Plant name	Thrombolytic assay	Hemolytic activity
EASF	90.21 ± 0.90	14.1 ± 1.01
Standard	97.32 ± 1.04	89.65 ± 1.10

*Note:* All experiments are performed in triplicate, and results are expressed as a percentage.

#### 3.2.5 Hemolytic Activity

Hemolytic activity of EASF is shown in Table 7. The activity observed in percentage is 14.1% ± 1.01%, which means no hemolytic effect is observed compared to the standard of 89.65% ± 1.10%.

### 3.3 In Silico Studies

#### 3.3.1 Molecular Docking

All GC–MS‐identified compounds were screened for α‐amylase, tyrosinase, and urease using molecular docking studies. Molecular docking studies showed five compounds bound more avidly than acarbose (standard) to α‐amylase. Stigmasterol was the most active of all the tested compounds and possessed an 8.7 binding affinity. It interacted in the active site of the α‐amylase as it formed a conventional hydrogen bond with the GLU 261 amino acid residue. 2D structures of ligands with α‐amylase show that kauren‐19‐oic acid and 17.beta.‐hydroxy‐5.alpha.‐androstane acetate showed H‐bond interactions. In contrast, isopimaric acid, methyl ester, and gamma‐sitosterol showed no H‐bond interactions. The docking results of tyrosinase showed that kauren‐19‐oic acid exhibits the highest binding affinity of −8.6, which interacts in the active site of tyrosinase by forming a conventional hydrogen bond interaction with GLN44, LYS180, and the amino acid residue. From the 2D structures of ligands with tyrosinase, it was clear that isopimaric acid, methyl ester, stigmasterol, and gamma‐sitosterol showed H‐bond interactions.

**FIGURE 3:**
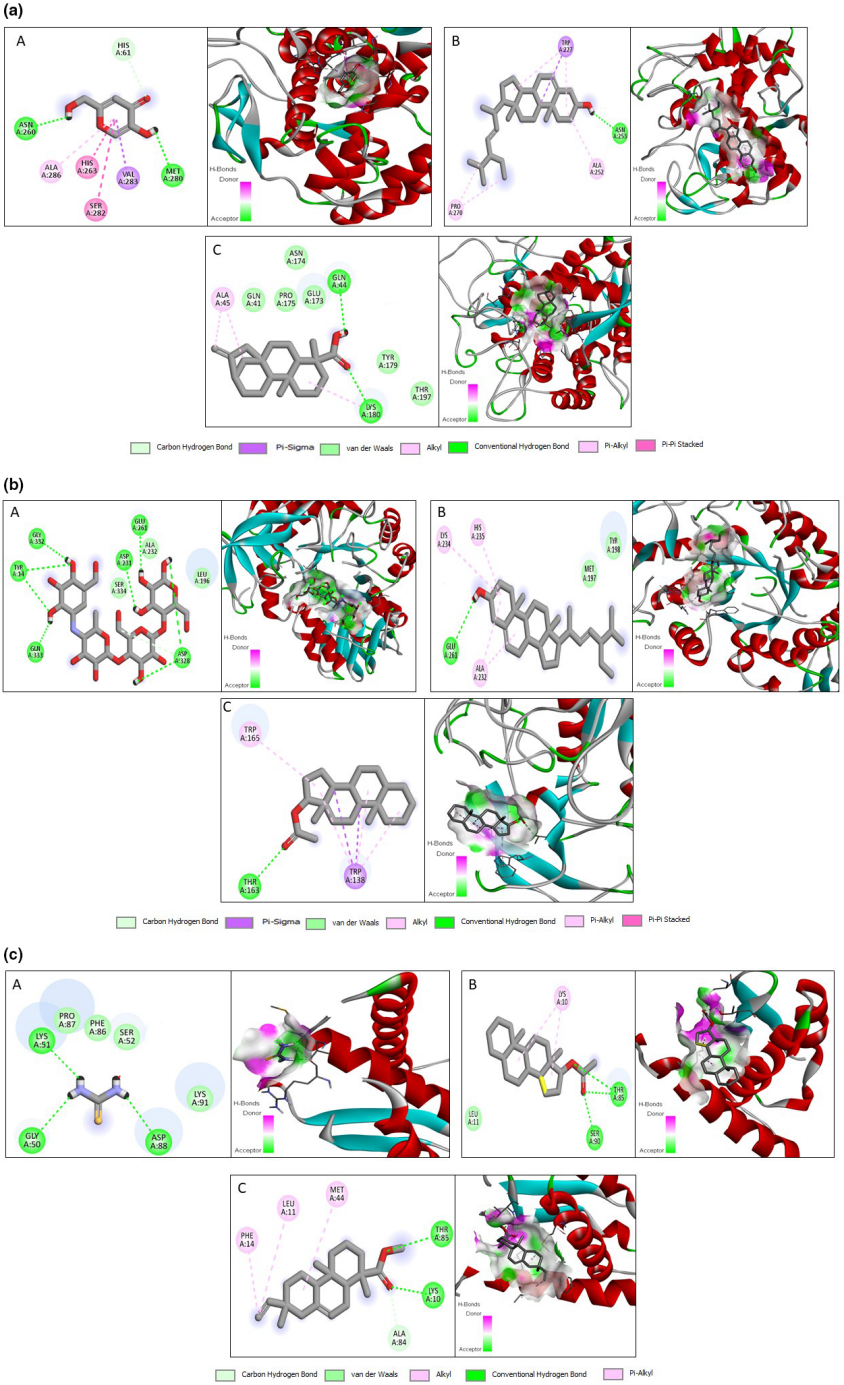
Figure 3a. 2D & 3D structure of the interaction of different compounds with tyrosinase enzyme. Where, (A) Kojic acid, (B) Stigmasterol, (C) Kauren‐19‐oic acid. Figure 3b. 2D & 3D structure of the interaction of different compounds with α‐amylase enzyme. Where, (A) Acarbose, (B) Stigmasterol, (C) 17.beta.‐Hydroxy‐5.alpha.‐androstane acetate. Figure 3c. 2D & 3D structure of the interaction of different compounds with the urease enzyme. Where, (A) Thiourea, (B) 17.beta.‐Hydroxy‐5.alpha.‐androstane acetate, (C) Isopimaric acid, methyl ester.

In contrast, 17. beta‐hydroxy‐5‐alpha‐androstane acetate did not show any H‐bond interactions. The docking results of urease showed that isopimaric acid, methyl ester, exhibits the highest binding affinity of −8, which interacted in the active site of urease by forming conventional hydrogen bond interactions with THR 85, LYS 10, amino acid residue, and carbon‐H‐Bond interactions with ALA 84 amino acid residue. The 2D structures of ligands with urease clearly showed Kauren‐19‐oic acid, 17 beta‐Hydroxy‐5‐alpha‐androstaneacetate, Isopimaric acid, methyl ester, Stigmasterol, and H‐bond interactions. In contrast, gamma‐sitosterol did not show any H‐bond interactions. The binding results of all phytocompounds identified by GC–MS of EASF against tyrosinase, alpha‐amylase, and urease enzymes are expressed in Table 8. 2D and 3D structures of the best docked compounds against tyrosinase, alpha‐amylase, and urease enzymes are represented in Figures 3a, 3b and 3c, respectively.

**TABLE 8:** Binding results of phytocompounds identified by GC–MS of EASF against tyrosinase, alpha‐amylase, and urease enzyme.

Peak no	Compound name	Tyrosinase	Alpha amylase	Urease
1	3(2H)‐Furanone, 4‐hydroxy‐5‐methyl‐	−4.9	−4.4	−4
2	2,4(1H,3H)‐Pyrimidinedione, 5‐methyl‐	−5.6	−5.1	−4.2
3	Oxopyrans	−5.5	−5.5	−4.3
4	L‐Sorbose	−5.5	−5.8	−4.4
5	5‐Hydroxymethylfurfural	−5.3	−4.5	−4.4
6	2‐Decenal, (E)‐	−4.6	−4.4	−4
7	Guanosine	−6.5	−7.1	−5.9
8	Beta‐Spathulenol	−7.7	−7	−6
9	Hexadecane	−4.1	−4.7	−4
10	1‐Decanamine	−4.3	−4.4	−3.8
11	Octadecane	−4.7	−4.3	−4.2
12	7,9‐Di‐tert‐butyl‐1‐oxaspiro (4,5) deca‐6,9‐diene‐2,8‐dione	−6.5	−6.9	−6.3
13	Hexadecanoic acid, methyl ester	−5	−4.7	−4.5
14	Hexadecanoic acid	−4.8	−4.8	−4.8
15	Hexadecanoic acid, ethyl ester	−4.8	−4.4	−4.6
16	ICOSANE	−4	−4.6	−4.1
17	9,12‐Octadecadienoic acid (Z, Z)‐, methyl ester	−4.5	−4.9	−4.5
18	9‐Octadecenoic acid, methyl ester, (E)‐	−5	−4.9	−4.6
19	Methyl stearate	−4.2	−4.4	−4.3
20	Oleic acid	−4.6	−4.7	−4.3
21	Linoleic acid ethyl ester	−4.2	−5.1	−4.7
22	9‐Octadecenoic acid (Z)‐, ethyl ester	−4.7	−4.6	−4.3
23	Octadecanoic acid, ethyl ester	−4.6	−4.6	−4.7
24	Docosane	−4.5	−4.5	−3.8
25	9‐Octadecenethioic acid,12‐hydroxy‐, S‐t‐butyl ester	−5.4	−5.1	−4.6
26	Kauren‐19‐oic acid	**−8.6**	−7.8	−7.3
27	17.beta.‐Hydroxy‐5.alpha.‐androstane acetate	−7.3	−8.4	−7.4
28	Agathic acid	−7.3	−7.2	−7
29	Isopimaric acid, methyl ester	−6.7	−7.7	−8
30	Tetrahydroaraucarolone	−6.7	−7.3	−6.4
31	Tetracosanoic acid, methyl ester	−4.3	−5	−4.2
32	1‐Undecene‐3,6‐dione, 1‐(2,6,6‐trimethyl1‐cyclohexen‐1‐yl)‐	−5.8	−5.8	−5.4
33	Stigmasterol	−8.1	−8.7	−7.5
34	Gamma‐Sitosterol	−7.2	−8.4	−6.6
35	Standard	−5.2^a^	−7.4^b^	−2.6^c^

^a^ Kojic acid.

^b^ Acarbose.

^c^ Thiourea.

#### 3.3.2 ADME Analysis

SWISS ADME online software was used for ADME analysis of the five phytoconstituents from GC–MS of EASF based on maximum binding affinity. This online tool details the physicochemical properties, pharmacokinetic, and drug‐likeness features of the respective phytoconstituents. If either the drug or phytoconstituents do not fulfill two or more rules of Lipinski's Rule of Five, the drug or phytoconstituents are considered non‐oral bioavailable.

**FIGURE 4:**
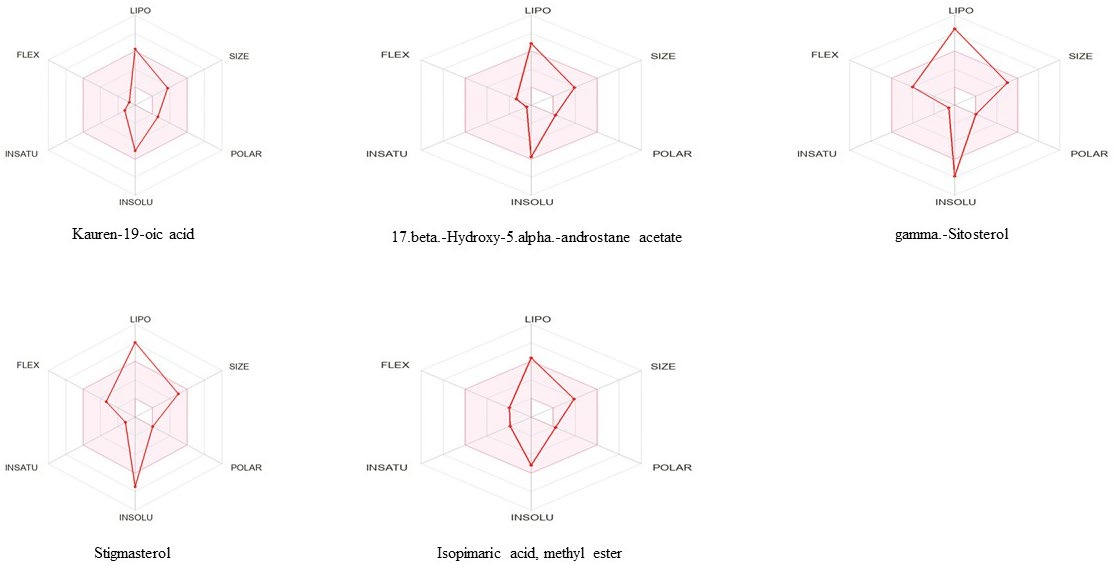
Bioavailability radar of phytocompounds exhibiting maximum binding results.

ADME of selected phytoconstituents of EASF showed that all selected phytoconstituents from EASF violate one rule (Described in Table 9). Each of the chosen phytoconstituents is appropriate for oral administration and has features of an orally active drug with drug‐like properties. The oral drug delivery system offers excellent safety, patient compliance, pain avoidance, and many other benefits over different routes in the drug administration system. Table 9 outlines the physicochemical properties, including molecular weight, lipophilicity, bond rotations, pharmacokinetic behavior, and hydrogen bond donor and acceptor of selected and analyzed phytoconstituents. Figure 4 shows the bioavailability radar of phytoconstituents from EASF. However, no literature is reported regarding ADME analysis of *A. squamosa* .

**TABLE 9:** ADME of best‐docked compounds.

SR NO	Best docked compounds	HBD	HBA	MWT	Lipophilicity	MR	LR
1	Kauren‐19‐oic acid	1	2	302.45	4.63	90.32	Yes, one violation
2	17.beta.‐Hydroxy‐5.alpha.‐androstane acetate	0	2	318.49	4.96	95.37	Yes, one violation
3	Isopimaric acid, methyl ester	0	2	316.48	4.76	96.28	Yes, one violation
4	Stigmasterol	1	1	412.69	6.62	132.7	Yes, one violation
5	Gamma.‐Sitosterol	1	1	414.71	6.73	133.2	Yes, one violation

Abbreviations: HBA, hydrogen bond acceptor; HBD, hydrogen bond donor; LR, Lipinski's rule; MR, molar refractivity; MWT, molecular weight.

#### 3.3.3 Toxicity Analysis

Five phytoconstituents with maximum binding affinity were selected for toxicity analysis using PROTOX online software from GC–MS of EASF. This web‐based tool may give information on the selected phytoconstituents about predicted LD50, toxicity class, predicted hepatotoxicity, predicted carcinogenicity, predicted mutagenicity, predicted immunotoxicity, and cytotoxicity.

All the phytoconstituents showed negative hepatotoxicity, carcinogenicity, mutagenicity, and cytotoxicity results. Only kauren‐19‐oic acid showed positive results in hepatotoxicity, and isopimaric acid, methyl ester, stigmasterol, and gamma‐sitosterol showed positive results in immunotoxicity. All the phytoconstituents fall in toxicity classes 4 and 5. Results of the toxicity evaluation of EASF are expressed in Table 10.

**TABLE 10:** Toxicity evaluation of EASF.

Sr no.	Compound name	Predicted LD_50_ (mg/kg)	Predicted toxicity class	Hepatotoxicity	Carcinogenicity	Mutage nicity	Immuno toxicity	Cytotoxicity
1	Kauren‐19‐oic acid	1000	4	+	−	−	−	−
2	17.Beta.‐Hydroxy‐5.alpha.‐androstane acetate	3100	5	−	−	−	−	−
3	Isopimaric acid, methyl ester	5000	5	−	−	−	+	−
4	Stigmasterol	890	4	−	−	−	+	−
5	gamma‐Sitosterol	890	4	−	−	−	+	−

*Note:* +, toxic; −, not toxic.

## 4 Discussion

Phytochemical analysis of the ethanolic extract of *Annona squamosa* fruit (EASF) revealed the presence of a diverse array of primary and secondary metabolites, including proteins, lipids, amino acids, carbohydrates, reducing sugars, cardiac glycosides, phenols, and alkaloids. These classes of phytochemicals are widely documented in the literature for their pharmacological properties such as antidiabetic, antibacterial, antifungal, anti‐inflammatory, antimicrobial, and antihypertensive activities. Specifically, alkaloids are known for their antifungal, antiviral, antitumor, and antimicrobial properties (Demir and Ağaoğlu 2021; Han et al. 2007; Tungmunnithum et al. 2018), while phenols and carbohydrates have been associated with antibacterial potential. Therefore, the medicinal attributes of EASF are likely attributable to the presence of these compounds. The total phenolic content (TPC) and total flavonoid content (TFC) were found to be relatively high in EASF, which is a notable finding considering that the leaves of *A. squamosa* are previously reported to have significantly elevated TPC and TFC levels (Nguyen et al. 2020). High TPC and TFC are strongly correlated with antioxidant potential (Aggarwal et al. 2018), underscoring the therapeutic significance of the fruit extract. Comprehensive phytochemical profiling using GC–MS analysis identified 34 distinct compounds in the EASF, with major constituents including kauren‐19‐oic acid, 17β‐hydroxy‐5α‐androstane acetate, isopimaric acid methyl ester, stigmasterol, and γ‐sitosterol. These compounds belong primarily to fatty acids, sterols, terpenoids, and esters, each known to exhibit a range of biological activities. Several identified metabolites have been reported in previous studies to possess antimicrobial, antitumor, antioxidant, anticancer, and anti‐inflammatory properties (Chandrasekaran et al. 2011; Pinto et al. 2017). The detection of these bioactive metabolites supports the therapeutic potential of EASF and provides a promising foundation for further studies on the isolation and characterization of novel lead compounds for drug development targeting infections, cancer, and inflammatory diseases.

The anti‐oxidant activity observed in the ABTS assay was notably higher than that reported for *A. squamosa* leaves in earlier studies (Nguyen et al. 2020). Since reactive oxygen species (ROS) and oxidative stress are known contributors to carcinogenesis and inflammation, natural antioxidants are critical in neutralizing oxidative damage to biomolecules such as DNA, proteins, and lipids. ROS‐induced DNA mutations, including those affecting tumor suppressor genes such as *p53*, are pivotal in the initiation and progression of various cancers (Shehata et al. 2021). Antioxidant, antibacterial, and anti‐inflammatory constituents in EASF also suggest potential dermatological applications. Tyrosinase, a copper‐containing enzyme involved in melanogenesis and enzymatic browning, is a key target for the cosmetic, pharmaceutical, and food industries. Tyrosinase inhibitors are used as skin depigmenting agents and antibrowning agents in food preservation. In this study, EASF exhibited notable tyrosinase inhibition (Table 5), outperforming *A. squamosa* leaf extract as reported in previous literature (Zolghadri et al. 2019). Standard assays employing substrates such as L‐tyrosine or L‐DOPA confirm the inhibitory potential through dopachrome formation. The results shown in this study (Chatatikun and Chiabchalard 2017) demonstrate that *A. squamosa* leaves extract shows less tyrosinase inhibitory activity than EASF.

Compounds tentatively identified by the GC–MS of EASF are reported as antidiabetic agents, that is, stigmasterol (Ashraf and Bhatti 2021) and gamma‐sitosterol (Sonia and Singh 2019). Despite the promising pharmacological profile, EASF did not exhibit significant antidiabetic activity compared to the seed extracts of *A. squamosa* , which have shown considerable efficacy in earlier studies (Sangala et al. 2011). Diabetes mellitus, a major metabolic disorder characterized by chronic hyperglycemia and insulin dysfunction, continues to pose a global health challenge. According to WHO estimates, the prevalence of diabetes may reach 300 million by 2030 (Sangala et al. 2011), underscoring the need for effective, plant‐based antidiabetic therapies.

*Helicobacter pylori* , a gram‐negative opportunistic pathogen, is implicated in peptic ulcers, gastric carcinoma, and mucosa‐associated lymphoid tissue (MALT) lymphomas. While nearly 50% of the global population is colonized by *H. pylori* , only about 20% develop clinical symptoms (Yogeswari et al. 2012). The increasing emergence of antibiotic‐resistant *Helicobacter pylori* strains has necessitated the search for novel, natural antimicrobial agents. In this context, the anti‐urease activity observed in EASF was notably high, surpassing standard references. Urease is critical for *H. pylori* colonization and pathogenesis by facilitating survival in acidic environments (Yogeswari et al. 2012). To the best of our knowledge, no prior study has reported the anti‐urease activity of *A. squamosa* , making this a novel finding with potential therapeutic implications. The antibacterial activity of EASF is likely attributable to alkaloids and phenolic compounds. The increasing resistance of bacteria to conventional antibiotics, exacerbated by geographic variations in antibiotic use, is a pressing global health concern. Resistance diminishes treatment efficacy and poses serious risks to patient safety (Salehi et al. 2018). Natural antimicrobial agents are being widely explored for their diverse mechanisms of action, including antibacterial, antiviral, and antifungal effects (Modolo et al. 2015). Traditionally, *A. squamosa* leaves have been used in antibacterial gels (Salehi et al. 2018). Phytoconstituents tentatively identified by GC–MS of EASF are previously reported as potent antimicrobial agents, that is, 3(2H)‐furanone, 4‐hydroxy‐5‐methyl (Schwab 2013), and hexadecane (Yogeswari et al. 2012), which supports the potential application of the EASF as an antibacterial agent. Cytotoxicity assessment through hemolytic activity offers insights into the safety profile of plant extracts.

Hemolysis, the rupture of erythrocyte membranes, releasing hemoglobin, can indicate the presence of harmful saponins or other bioactive constituents (Kumar et al. 2011). EASF demonstrated hemolytic activity at 14.1% ± 1.01%, below the critical threshold of 30% considered toxic (Sowemimo‐Coker 2002), indicating that the extract is safe for human use. Thrombolytic activity, referring to the lysis of blood clots, is vital in managing cardiovascular conditions. Conventional thrombolytic agents such as tissue plasminogen activator and streptokinase are widely used but are associated with adverse effects and high costs. EASF exhibited potent thrombolytic activity (85.5%), suggesting its potential as a natural, cost‐effective alternative to synthetic thrombolytics.

In silico molecular docking studies were conducted to evaluate the binding affinities of GC–MS‐identified phytoconstituents with urease, α‐amylase, and tyrosinase. Key interactions included hydrogen bonding and hydrophobic interactions (e.g., van der Waals, alkyl, and π‐alkyl), essential in protein‐ligand stability. The top five compounds based on docking scores were further assessed using SwissADME and ProTox‐II web tools to predict pharmacokinetics and toxicity profiles. According to Lipinski's Rule of Five, a compound is considered a poor candidate for oral administration if it violates two or more rules, which include molecular weight, lipophilicity (LogP), hydrogen bond donors, molar refractivity, and hydrogen bond acceptors (Aati et al. 2022). After evaluation, all five compounds showed only one violation, indicating good oral bioavailability. All selected compounds exhibited acceptable toxicity parameters, falling within Class 4 and 5 toxicity levels. Notably, kauren‐19‐oic acid was flagged for potential hepatotoxicity, while isopimaric acid methyl ester, stigmasterol, and γ‐sitosterol showed immunotoxic effects. However, none of the compounds were predicted to be carcinogenic, mutagenic, or cytotoxic. No toxicity assessment of *A. squamosa* fruit compounds has been reported in the literature, making this the first study to offer such insights. These findings support the therapeutic potential of EASF, highlighting the importance of further isolation and evaluation of its bioactive compounds for drug development.

## 5 Conclusions

This study highlights the phytochemical richness and broad biological potential of the Saudi cultivar of *Annona squamosa* . The ethanolic extract (EASF) demonstrated a high content of polyphenols, flavonoids, and tannins, as confirmed by phytochemical analysis and GC–MS profiling. The extract exhibited strong anti‐oxidant capacity, significant thrombolytic activity, and a low hemolytic effect. Noteworthy antibacterial activity was observed against *Staphylococcus epidermidis* , and the extract effectively inhibited key clinical enzymes, including urease, tyrosinase, and α‐amylase. Molecular docking and ADMET analysis of selected phytocompounds further validated their potential therapeutic relevance. Collectively, these findings support the use of the Saudi sugar apple cultivar as a functional food with pharmaceutical applications and highlight its promise for future development in the nutraceutical industry. Further in vivo studies, including acute and sub‐acute toxicity evaluations, are recommended to establish its safety profile.

## Author Contributions

**Hanan Y. Aati:** conceptualization (equal), resources (equal), supervision (equal). **Renad Al‐Arifi:** formal analysis (equal), methodology (equal). **Chitchamai Ovatlarnporn:** investigation (equal), methodology (equal), project administration (equal), software (equal). **Khloud AlYami:** formal analysis – equal, methodology – equal, reviewing and editing – equal. **Abdul Rauf:** formal analysis (equal), writing – original draft (equal). **Abdul Basit:** investigation (equal), methodology (equal), software (equal), writing – original draft (equal). **Huma Rao:** formal analysis (equal), software (equal). **Maria Batool:** data curation (equal), software (equal). **Kashif ur Rehman Khan:** conceptualization (equal), investigation (equal), supervision (equal), writing – review and editing (equal).

## Conflicts of Interest

The authors declare no conflicts of interest.

## Data Availability

Data will be made available upon request.
