# Correction to “GC–MS Profiling and In Vitro and Silico Evaluation of Biological Propensities of Saudi Cultivar of Sugar Apple (*Annona squamosa* L.): A Preliminary Multidimensional Approach for the Development of Nutraceuticals”

**DOI:** 10.1002/fsn3.71111

**Published:** 2025-10-24

Aati, H. Y., R. Al‐Arifi, C. Ovatlarnporn, et al. 2025. “GC–MS Profiling and In Vitro and Silico Evaluation of Biological Propensities of Saudi Cultivar of Sugar Apple (Annona squamosa L.): A Preliminary Multidimensional Approach for the Development of Nutraceuticals.” Food Science & Nutrition 13, no. 9: e70723. https://doi.org/10.1002/fsn3.70723.

In the published version, there was an error in the funding and acknowledgment. The correct statement is:

The authors thank the Ongoing Research Funding Program (ORF‐2025‐504), King Saud University, Riyadh, Saudi Arabia.

The online version has been corrected accordingly.

We apologize for this error.

